# Prognostic Role of M6A-Associated Immune Genes and Cluster-Related Tumor Microenvironment Analysis: A Multi-Omics Practice in Stomach Adenocarcinoma

**DOI:** 10.3389/fcell.2022.935135

**Published:** 2022-06-24

**Authors:** Na Luo, Min Fu, Yiling Zhang, Xiaoyu Li, Wenjun Zhu, Feng Yang, Ziqi Chen, Qi Mei, Xiaohong Peng, Lulu Shen, Yuanyuan Zhang, Qianxia Li, Guangyuan Hu

**Affiliations:** ^1^ Department of Oncology, Tongji Hospital, Tongji Medical College, Huazhong University of Science and Technology, Wuhan, China; ^2^ Department of Obstetrics and Gynecology, Union Hospital, Tongji Medical College, Huazhong University of Science and Technology, Wuhan, China

**Keywords:** prognosis, bioinformatic analysis, M6A, survival, tumor microenvironment, stomach adenocarcinoma

## Abstract

N6-methylandrostenedione (m6A) methylation plays a very important role in the development of malignant tumors. The immune system is the key point in the progression of tumors, particularly in terms of tumor treatment and drug resistance. Tumor immunotherapy has now become a hot spot and a new approach for tumor treatment. However, as far as the stomach adenocarcinoma (STAD) is concerned, the in-depth research is still a gap in the m6A-associated immune markers. The Cancer Genome Atlas (TCGA) and Gene Expression Omnibus (GEO) databases is extremely important for our research, where we obtained gene mutation, gene expression data and relevant clinical information of STAD patients. Firstly, the samples from GEO were used as external validation groups, while the TCGA samples were divided into a training group and an internal validation group randomly. Using the way of Single factor COX-LASSO- and multi-factor Cox to construct the prognostic model. Then, all samples were subjected to cluster analysis to generate high and low expression groups of immune gene. Meanwhile, we also collected the correlation between these types and tumor microenvironment. On this basis, a web version of the dynamic nomogram APP was developed. In addition, we performed microenvironmental correlation, copy number variation and mutation analyses for model genes. The prognostic model for STAD developed here demonstrated a very strong predictive ability. The results of cluster analysis manifested that the immune gene low expression group had lower survival rate and higher degree of immune infiltration. Therefore, the immune gene low expression group was associated with lower survival rates and a higher degree of immune infiltration. Gene set enrichment analysis suggested that the potential mechanism might be related to the activation of immunosuppressive functions and multiple signaling pathways. Correspondingly, the web version of the dynamic nomogram APP produced by the DynNom package has successfully achieved rapid and accurate calculation of patient survival rates. Finally, the multi-omics analysis of model genes further enriched the research content. Interference of RAB19 was confirmed to facilitate migration of STAD cells *in vitro*, while its overexpression inhibited these features. The prognostic model for STAD constructed in this study is accurate and efficient based on multi-omics analysis and experimental validation. Additionally, the results of the correlation analysis between the tumor microenvironment and m6Ascore are the basics of further exploration of the pathophysiological mechanism in STAD.

## Introduction

Stomach adenocarcinoma (STAD) is one of the leading malignancies contributing to the health burden as well as being the third leading cause of cancer deaths worldwide (Bray et al., 2018; Ferlay et al., 2019). Surgery is the main curative treatment for gastric cancer. However, as 80–90% of gastric cancer patients are already in the advanced stage at initial diagnosis, that’s almost impossible to cure (Wagner et al., 2010; Smyth et al., 2020). In recent years, with significant advances in new therapeutic strategies including chemotherapeutic agents, novel targeted agents and immunotherapeutic agents for patients with STAD, the overall efficacy and survival have improved, but the overall survival of patients is still not optimistic. As not all patients are responding to existing therapies based on recognized biomarkers (Chau, 2017; Song et al., 2017). Therefore, the further exploration of new markers which is leading to poor prognosis of STAD, classifying patients with different prognosis and enhancing the accuracy of predicting patient prognosis, is a positive significance to further improve early screening and treatment of STAD.

The study of N6-methyladenosine (m6A) has become more popular in recent years, leading to an increasing understanding on it. The N6-methyladenosine (m6A) methylation process includes three major methylation enzymes: methyltransferase (Writer), demethylase (Eraser), and methylation resolution protein (Reader) (Dominissini et al., 2012; Tong et al., 2018). M6A is involved in the regulation of genes at the transcriptional and post-transcriptional levels, which is necessary for cell survival and functional integrity (Yadav et al., 2018). Furthermore, m6A can be involved in tumorigenesis by regulating the expression of the tumor-associated genes BRD4, MYC, SOCS2, and EGFR (Vu et al., 2017; Yan et al., 2018; He et al., 2019; Lin et al., 2019). N6-methylandenosine (m6A) RNA methylation is linked to tumorigenesis and progression, which is regulated dynamically by m6A RNA methylation regulators (Weng et al., 2018; Zaccara et al., 2019; Wang et al., 2020a; Wang et al., 2020b; Chokkalla et al., 2020; Zhang et al., 2020). Studies have shown that there is a correlation between m6A and prognosis in STAD patients, and the m6A status is significantly connected to STAD tumor onset and progression. Li et al. have shown that the expression of FTO and ALKBH1 correlates with prognosis in STAD patients based on TCGA database exploration (Li et al., 2019). BATF2 regulates STAD progression *via* the METTL3, which provides a potential prognostic and therapeutic target for STAD therapy (Xie et al., 2020). METTL3 promotes angiogenesis and increases glycolysis through activation the AKT signaling pathway (Wang et al., 2020c). HBXIP exerts a pro-cancer cell invasion role through METTL3-mediated modification of MYC mRNA m6A (Yang et al., 2020). Knockdown of METTL14 promotes STAD malignant progression *via* activation of the Wnt/PI3K-AKT axis (Zhang et al., 2019). In addition, the characteristics of m6A RNA methylation for risk stratification can be utilized as an index of independent prognosis for overall survival (OS) in STAD (Mo et al., 2020). Additionally, PD1/PD-L1 checkpoint blockade is controlled by the m6A reader YTHDF1 and eraser FTO. Therefore, m6A modulators may be promising sites for anti-cancer immunotherapy (Han et al., 2019; Yang et al., 2019). Moreover, high anti-PD-1/L1 immunotherapy response and elevated neoantigen load was associated with a decreased m6Ascore in STAD patients (Zhang et al., 2020). In consequence, it is of great significance to study the role of m6A-regulated genes and their related pathways in immunotherapy for improving the efficacy and survival rate of immunotherapy in patients with STAD.

With the development of immunotherapy, various elements of the immune system including the immune microenvironment of tumor, immune-related genes, and immune cells have been found to play an important roles in the pathophysiology of tumors (O'Donnell et al., 2019; Atsavapranee et al., 2021; El-Mayta et al., 2021). The study of immunotherapy in STAD has attracted considerable increasing attention. Molecular characterization of STAD analyzed by the TCGA research network has shown increased expression of PD-L1 in a subpopulation of EBVs that account for 15% of STAD tumors (Comprehensive molecular characterization of gastric, 2014). In addition, it was shown that PD-L1 is expressed in both STAD cells and immune stroma. Surprisingly, Elizabeth et al. showed that increased CD8 infiltration was associated with shorter OS in STAD patients, and suggesting that adaptive immune tolerance mechanisms may be occurring (Thompson et al., 2017). Nivolumab and pembrolizumab are available for advanced gastric cancer patients (Noguchi et al., 2000; Janjigian et al., 2020). HER2-directed antibody-drug coupling, Disitamab vedotin (RC48), significantly enhanced antitumor response when combined with PD-1/PD-L1 immune checkpoint inhibitors, which was concomitant with immunomarker activation and substantial T-cell infiltration (Huang et al., 2022). Therefore, significant therapeutic advantages and clinical benefits may emerge from the combination with immunotherapy in the treatment of gastric adenocarcinoma in the future.

Research showed that m6A plays an essential role in regulating and modifing viral RNA expression. At the same time, m6Ascores have been used to forecast the effect of anti-PD-1/L1 immunotherapy. M6A is thus a reliable biomarker for both prognosis and evaluation of clinical response to immunotherapy (Zhang et al., 2020; Jin et al., 2021).

After downloading multiple public datasets, we used single-factor Cox regression, LASSO, and multi-factor Cox regression analyses to build a prognostic model with the basis of m6A-associated immune-related genes, and successfully created a web version of the dynamic nomogram APP (Geng et al., 2020). Then, the correlations between the two types and the tumor microenvironment and m6Ascore were calculated. Additionally, the findings of enrichment analysis further revealed the potential immunosuppressive mechanism. Further, we performed correlation analysis of immune subtypes, clinical stage, as well as the microenvironment for model genes. Finally, the biological role of RAB19 in STAD cells was revealed.

## Methods

### Data Collection and Variance Analysis

The gene expression dataset (HTSeq-FPKM), clinical information, and data related to mutation experiments for stomach adenocarcinoma (STAD) were obtained from The Cancer Genome Atlas (TCGA). The platform file (GPL6947-13512) and the STAD probe matrix file (GSE84433_series_matrix) were also downloaded from the GEO website as an external validation set. M6A regulatory genes were obtained from previously published literature (Xu et al., 2020). Here, we have extracted the immune-related genes (IRGs), which were contained in the Gene Set Enrichment Analysis (GSEA). All data were pretreated using the limma and sva packages. All samples were obtained via surgical excision of tissues from primary stomach adenocarcinoma patients.

First, we figured out the correlations between immune genes and m6A-regulated genes by using wilcox. test function and screened the results to obtain m6A-associated immune genes (*p*-value < 0.05 and |correlation coefficient| > 0.4) for subsequent analyses. Next, the corresponding hazard ratio (HR) values and *p*-values were obtained after survival analysis for m6A-regulated genes with univariate Cox analysis and the Kaplan-Meier method. An m6A prognostic network and co-expression network for m6A-regulated genesand immune genes were plotted using the igraph package (|correlation coefficient| > 0.6). Finally, the R package—limma was regarded as a tool to identify differential expression m6A-associated immune genes between normal and tumor groups [false discovery rate (FDR) < 0.05 and |log fold change (FC)| > 0.5]; the results are presented in the form of a heat map.

### Construction of Prognostic Model, Mutation Analysis, and Dynamic Nomogram

We randomly divided TCGA-STAD samples into two groups for training and internal validation, while corresponding GEO samples were used as external validation group. In the training group, we used the classical single variable cox model for regression, LASSO algorithm, and multiple variable Cox stepwise regression analyses to obtain the m6A-associated immune genes that had statistically significant effects on prognosis (*p* < 0.05). Moreover, in order to optimize the prognostic model, we removed highly correlated genes. Based on the median risk score of the training group as the cutoff value, all samples were separated into two groups, the high risk group and the low risk group, and to test the predictive capability of this model, our authors plotted survival curves and receiver operating characteristic (ROC) curves.

After the construction of the prognostic model, we visualized mutation waterfall plots by using the Maftools package for the high-risk and low-risk groups. Next, in the two groups, the effects of mutational load on survival were calculated.

The clinical translation value of our prognostic model will be enhanced by the download of the DynNom package, and the package exists can help us create the corresponding web-based dynamic column chart APP that allows rapid and accurate determination of patient prognosis.

### ssGSEA Analysis and Cluster Typing

For TCGA and GEO samples, in each sample, ssGSEA was used to calculate the contents of 23 immune cells using the GSVA package. All samples were clustered separately *via* using the Consensus Cluster Plus package based on the m6A-associated immune genes that affect prognosis (*p* < 0.05) to generate groups with high immune gene expression and low these gene expression. Based on the groups’ results, heat maps, violin plots, and microenvironmental survival curves of tumors were plotted for the two immune gene expression groups to visualize the correlation between m6A-associated immune genes and immunity. The org. Hs.eg.db R package was used to plot multi-GSEA enrichment curves. And the curves were based on five GO terms (gene ontology) of significant enrichment for the immune gene high-expression group, which was compared with the immune gene low-expression group, and Kyoto Encyclopedia of Genes and Genomes (KEGG) pathways (*p* < 0.05).

### Comprehensive Analysis for Model Genes

Firstly, we determined the correlations of individual model genes with immune subtypes, clinical stage, stem cell indices, and tumor microenvironment parameters. Furthermore, for model genes, the Tumor Immune Estimation Resource (TIMER) databases were used to calculate and analyze the copy number variation (CNV) frequency levels and the relationship between the immune cell infiltration of individual cells levels and the CNV. Finally, the mutation frequencies and structural domain mutations of model genes was downloaded from the cBioportal databases.

### Drug Sensitivity Analysis

To investigate the effect of prognostic models on the sensitivity of drugs, two sets of data, FDA-approved drug sensitivity-related and transcriptomic data were obtained from a database, which is called CellMiner database. The related link is https://discover.nci.nih.gov/cellminer/. In order to analyze gene expression and drug sensitivity, the Pearson correlation test was applied. Subsequently, the association with low-risk and high-risk populations in our prognostic model, as well as STAD-related drugs was analyzed using the “pROphetic” R package, and box plots were drawn.

### Cell Lines and siRNA and Plasmid Transfection

GES one Normal Gastric Epithelium Cell Line, STAD cell lines SUN-216, SGC-7901, AGS, N87, BGC-823 and HGC-27 were purchased from The American Type Culture Collection, which is also known as the ATCC. And RPMI 1640 medium (HyClone, United States) was used to culture these cell lines, along with 10% fetal bovine serum (FBS, Gibco, United States) in 5% CO2. Equally important, all of them are at 37°C. RiboBio (Guangzhou, China) synthesized SiRNA and pcDNA3.1-RAB19. And as described in the production instructions, they were successfully transfected into our target with HighGene plus transfection reagent, which is bought from Abconlal, Wuhan, China.

### qRT-PCR

The reverse transcription was performed by Hi Script II QRT SuperMix (Vazyme, China), and the RNA extraction from our cells using the TRIzol reagent (TaKaRa, Japan). Just like any other lab, we utilized Vazyme’s ChamQ Universal SYBR qPCR Master Mix (China) to perform qRT-PCR. The RAB19’s primer sequences were 5′- GTCCATCCCTCACTGGATTCA-3′(forward) and 5′-GCA​TCC​TCG​AAC​AGG​ACG​TG-3′ (reverse), and the primers sequences for the internal reference gene β-actin were 5′- GAC​AGT​CAG​CCG​CAT​CTT​CT -3′ (forward) and 5′- GCG​CCC​AAT​ACG​ACC​AAA​TC -3′ (reverse). Here, we set β-actin as an internal control for this study.

### Cell Counting Kit-8 Viability Detection

Cells (3,000 cells/well) were seeded in 96-well plates overnight for attachment. Then 100 µl per well FBS- free medium with 10% CCK8 (Bio-sharp, Hefei, China) was used instead incubating the cells for 1 h at 37°C. Microplate reader (BioTek, United States) was adopted to detect the OD values at 450 nm. These steps mentioned above were repeated at 0, 24, 48, 72, 96, and 120 h. Based on the OD values at 0 h, the relative absorbance was calculated.

### Wound Healing Assay and Transwell for Migration Assay

In 24-well plates, we used serum-free RPMI 1640 medium to inoculate the cells. The scratch experiments were prepared when the cells were grown to 100% density, then utilized 10 µl pipette tip**s** to create scratch wound**s**. The next, we took the images we need at 0, 24, and 48 h, which the area of wounds was quantified by ImageJ software. The transwell migration assay was described as follows: 4 × 10^4^ cells and 200 µl of serum-free culture medium were seeded on the upper transwell chambers, and in the lower chambers, there was 500 µl medium, that contained 20% FBS. It then took 20 h to incubate, after we relied on methanol to fix the cells which had migrated through membranes. For better counting under the light microscope, we added a staining step using 1% crystal violet as well.

### Statistical Analyses

Except when publicly available databases and special software were used, the R software (version 4.0.3) helped us to perform graphical plots and general routine statistical analyses. Here, our prognostic model was constructed by some classical methods such as LASSO algorithm, univariate Cox regression, and multifactor Cox stepwise regression analysis method *p* < 0.05 was a signal that was considered to indicate statistical significance. Two-tailed Students’ t-test was used to analyze the difference between two subgroups in analysis of experiment results.

## Results

### Correlation Statistics and Differences Analysis

**FIGURE 1:**
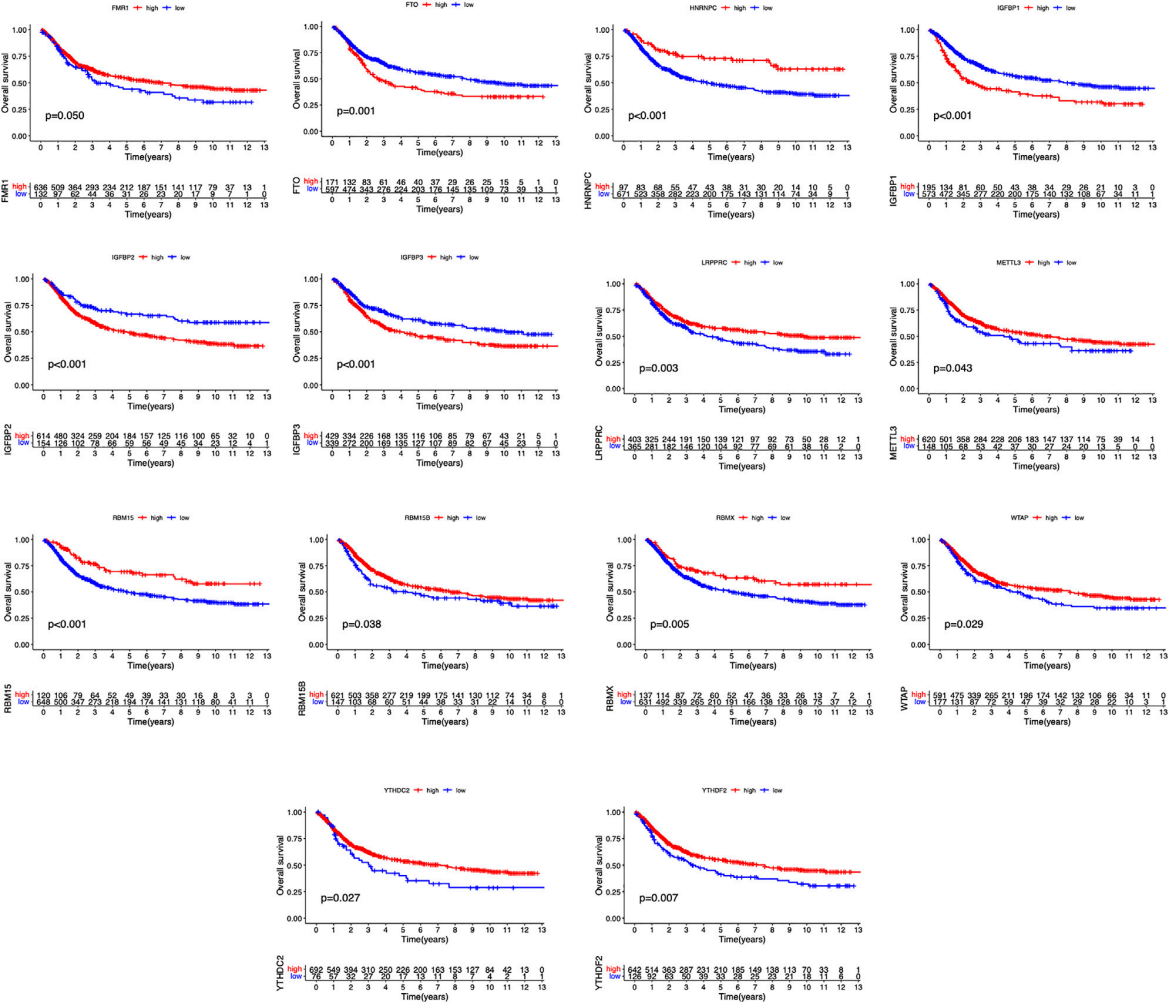
Survival curves of m6A regulatory genes in STAD.

**FIGURE 2:**
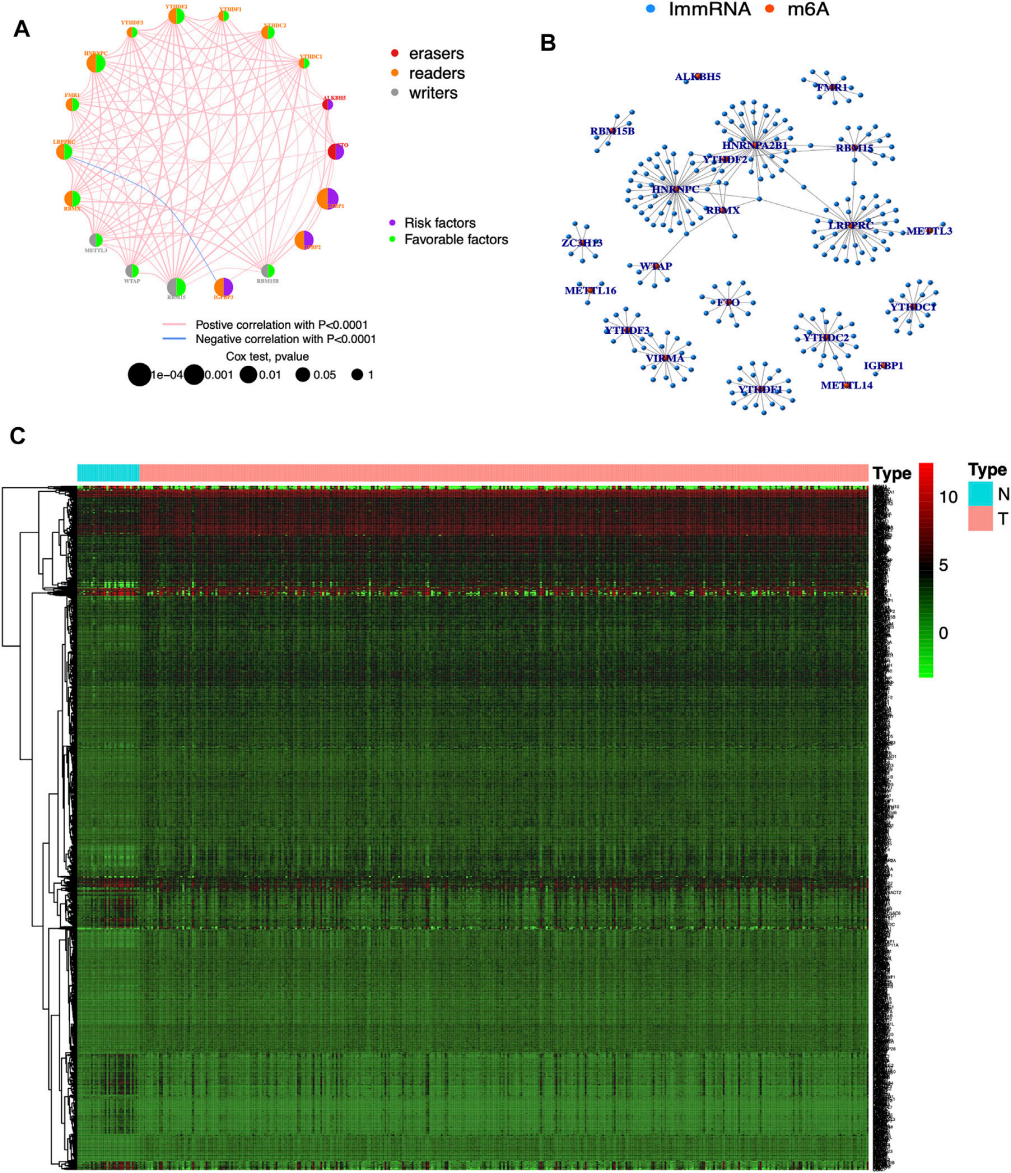
Visualization of m6A regulatory and immune genes **(A)** Prognostic network diagram of m6A. Nodes represent m6A regulatory genes, blue signifies negative co-expression relationships and red signifies positive co-expression relationships **(B)** Co-expression network of m6A regulatory and immune genes. Blue nodes signify immune genes, red nodes signify m6A regulatory genes, and connecting lines represent co-expression relationships **(C)** Heat map. N represents normal groups, T represents tumor groups. Green, black and red represent low, medium and high expressions respectively.

The clinical information corresponding to TCGA samples is presented in Table 1, and the results of survival analysis of m6A regulatory genes have been uploaded as Supplementary File S1. Also, the corresponding survival curves are shown in Figure 1. Among them, IGFBP1, IGFBP2, IGFBP3, and FTO were high-risk prognostic factors for STAD. METTL3, WTAP, RBM15, RBM15B, YTHDC2, YTHDF2, HNRNPC, FMR1, LRPPRC, and RBMX were low-risk prognostic factors for STAD. The m6A prognostic network graph clearly showed co-expression relationships among most of the m6A regulatory genes, and positive correlations were predominant (Figure 2A). Similarly, in the co-expression network of m6A regulatory genes and immune genes (|correlation coefficient| > 0.6), HNRNPC, HNRNPA2B1, and LRPPRC had highly correlated co-expression relationships with most of the immune genes (Figure 2B). Finally, the heat map clearly showed the m6A-associated immune genes that were differentially expressed between the two groups (|logFC| > 0.5) & FDR <0.05 (Figure 2C).

**TABLE 1:** Clinical characteristics of STAD patients in the two databases.

Characteristics		TCGA		GEO1		GEO2	
		Total	%	Total	%	Total	%
All		338	100.00	357	100.00	76	100.00
Age (y)	≥65	189	55.92	128	35.85	38	50.00
	<65	149	44.08	229	64.15	38	50.00
Gender	Male	216	63.91	242	67.79	54	71.05
	Female	122	36.09	115	32.21	22	28.95
Grade	G1	7	2.07	-	-	-	-
	G2	116	34.32	-	-	-	-
	G3	215	63.61	-	-	-	-
	G4	0	0.00	-	-	-	-
Stage	I	42	12.43	-	-	-	-
	II	112	33.14	-	-	-	-
	III	153	45.27	-	-	-	-
	IV	31	9.17	-	-	-	-
T stage	T1	15	4.44	11	3.08	0	0.00
	T2	72	21.30	35	9.80	3	3.95
	T3	167	49.41	67	18.77	25	32.89
	T4	84	24.85	244	68.35	48	63.16
M stage	M0	319	94.38	-	-	-	-
	M1	19	5.62	-	-	-	-
N stage	N0	104	30.77	71	19.89	9	11.84
	N1	95	28.11	155	43.42	33	43.42
	N2	71	21.01	99	27.73	33	43.42
	N3	68	20.12	32	8.96	1	1.32

### The Prognostic Model, Mutation Results, and Dynamic Nomogram

**FIGURE 3:**
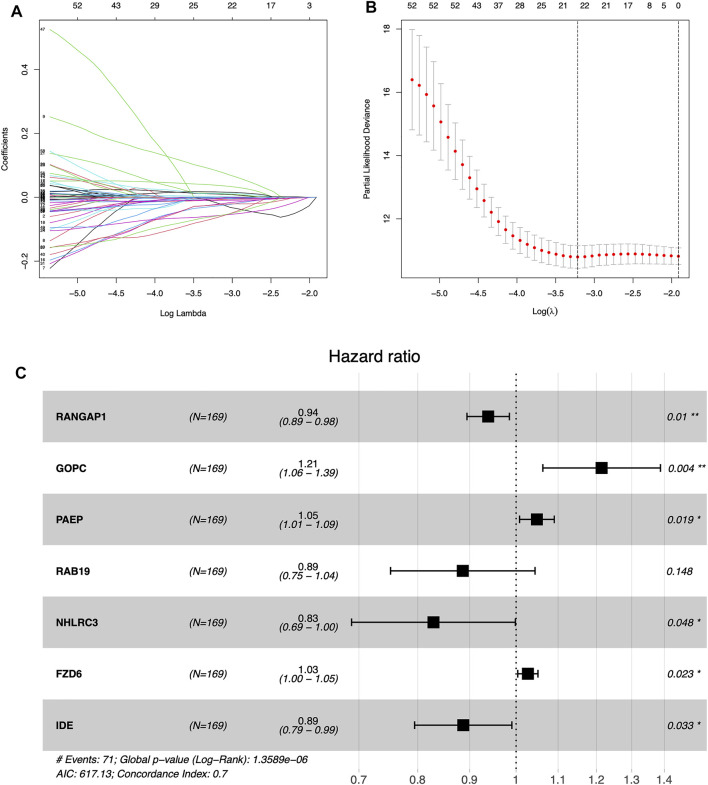
Establishing the prognostic model by LASSO regression analysis **(A)** LASSO coefficient plot **(B)** The training group containing the best log lambda value **(C)** Forest map showing 95% confidence intervals and HR values.

Supplementary File S2 (containing training group), Supplementary File S3 (containing internal validation group) and Supplementary Files S4,5 (containing external validation group) are available as supplementary materials. We obtained the results of the univariate Cox analysis as follows. There were totals of 64 m6A-associated immune genes affecting prognosis. Using LASSO algorithms to help us remove highly correlated genes was extremely reliable, and after that we needed to build prognostic models using multivariate Cox stepwise regression, and for every participant we calculated a risk score as follows: risk Score = (−0.063)*RANGAP1 + 0.194*GOPC +0.047*PAEP + (−0.121)*RAB19 + (−0.188)*NHLRC3 + 0.027*FZD6 + (−0.120)*IDE (Figures 3A–C; Table 2).

**TABLE 2:** the result of Multivariate COX regression analysis.

Id	Coef	HR	HR.95L	HR.95H	Pvalue
RANGAP1	−0.0633837	0.93858324	0.89445725	0.98488608	0.00988514
GOPC	0.19413485	1.21426002	1.06250641	1.387688	0.00437086
PAEP	0.04719467	1.04832607	1.0078499	1.09042779	0.01881494
RAB19	−0.1209739	0.88605707	0.7520692	1.04391607	0.14812852
NHLRC3	−0.1875121	0.82901909	0.68840239	0.99835891	0.04800963
FZD6	0.02671131	1.02707126	1.00377194	1.05091139	0.02251669
IDE	−0.1199501	0.8869647	0.79426786	0.99047995	0.03318732

**FIGURE 4:**
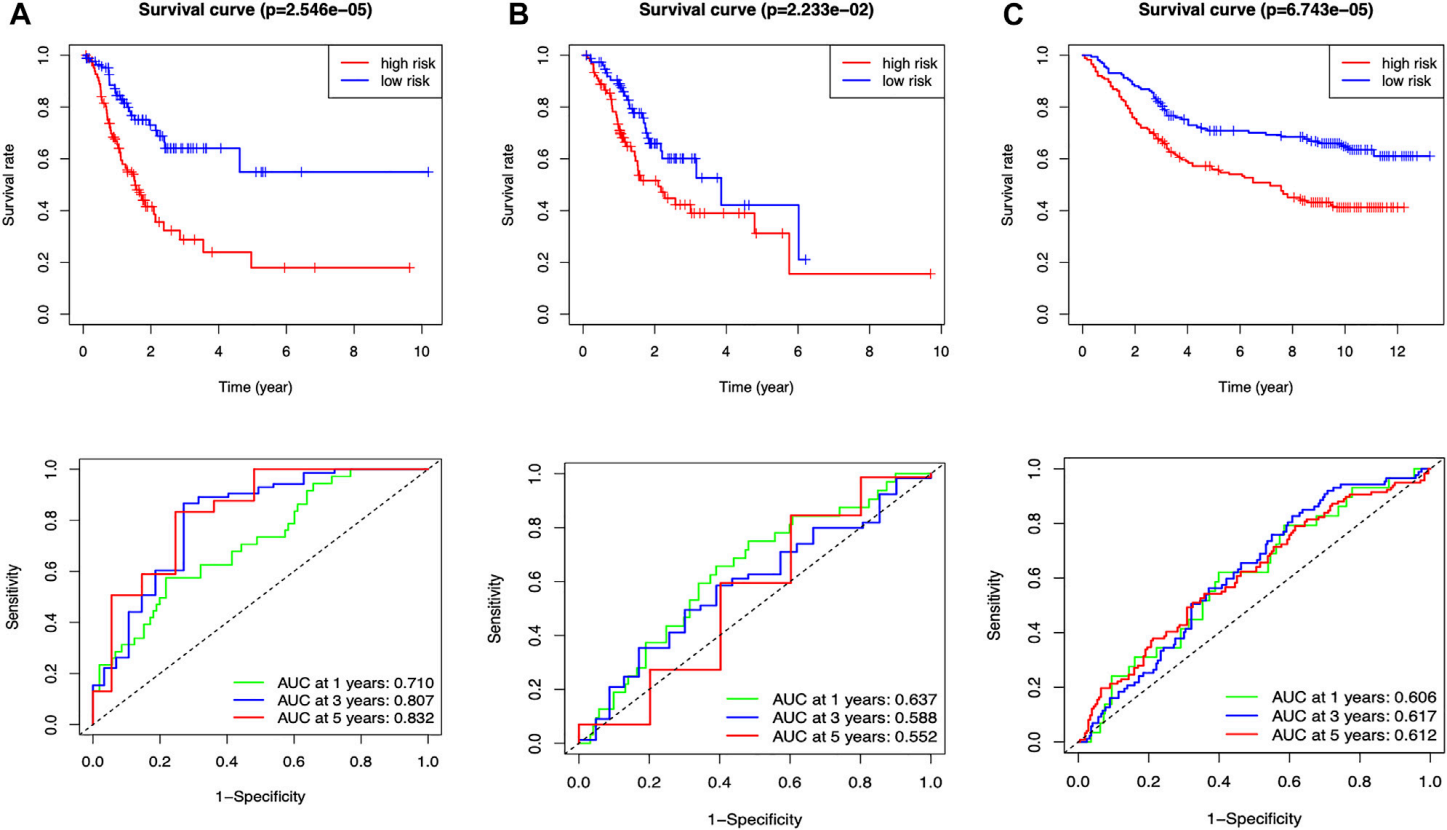
Clinical prognostic model evaluation **(A)** Training group **(B)** Internal validation group **(C)** External validation group, GSE84433_series_matrix.

The median risk assessment was generated in the training group, and then the samples of the training groups, internal validation groups and external validation groups were subdivided into high and low-risk groups. Survival rates are variable, with the low-risk group being significantly higher than the high-risk group in all groups. The area under the ROC curve revealed extremely high predictive efficacy of our constructed prognostic model (Figure 4).

**FIGURE 5:**
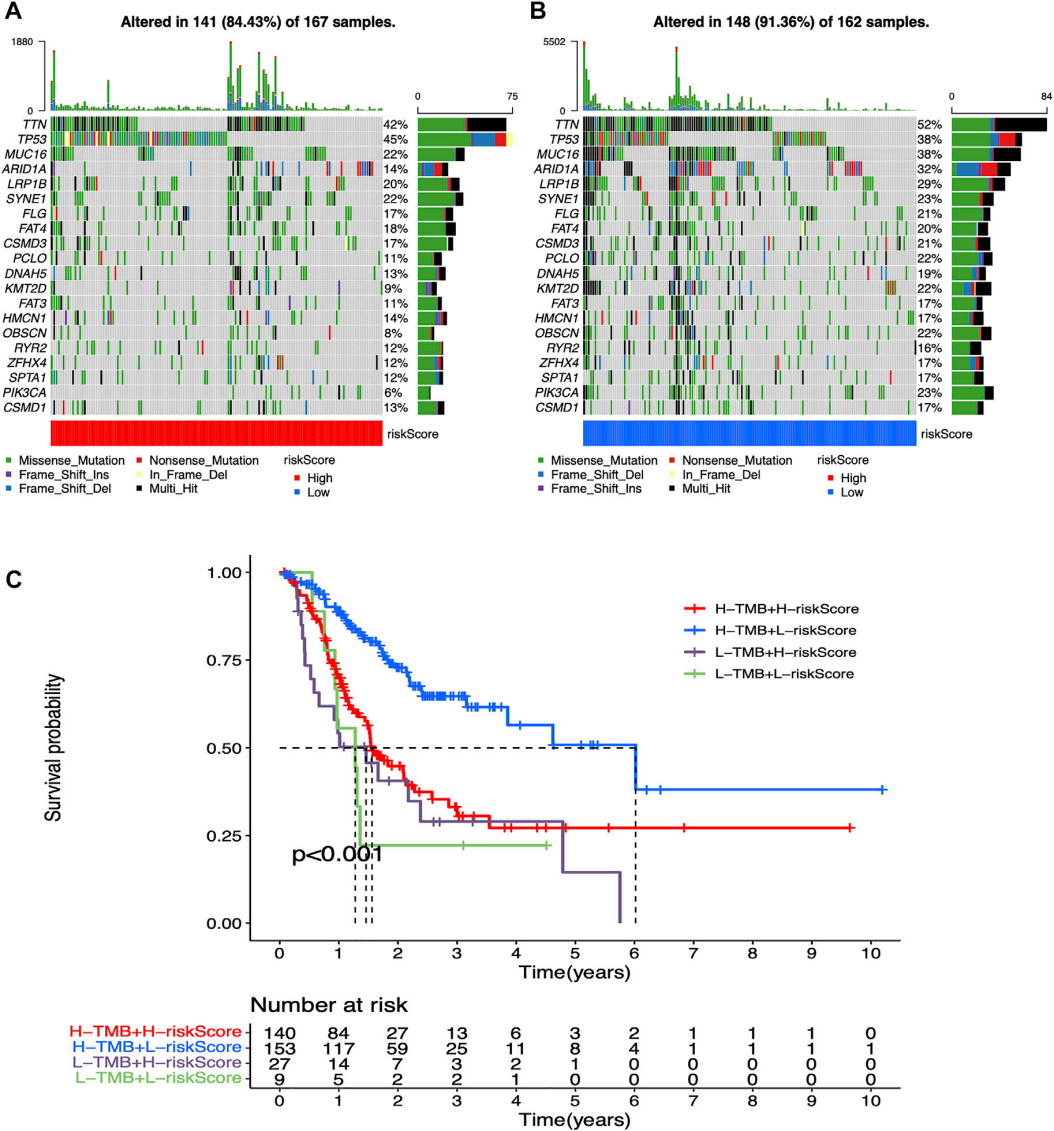
Mutation analysis for low-risk and high-risk groups **(A)** Mutation waterfall plot in high-risk participants. Mutation frequencies are represented on the right **(B)** Mutation waterfall plot in low-risk participants. Mutation frequencies are represented on the right **(C)** Survival analysis of mutation load combined with risk values.

Mutation waterfall plots for both two groups intuitively showed that the genes, which own the highest mutation frequencies in both two risk groups were predominantly TTN, TP53, and MUC16 (Figures 5A,B). In addition, mutational load significantly affected the survival rate in participant in both two groups, enabling further refined stratification of prognosis (Figure 5C).

We created a web-based version of the Dynamic Column Plot APP (available at https://u20131050.shinyapps.io/STAD-m6A_ImmRNA-Dynamic_nomogram/) to enable direct online calculation of patient survival by entering the expression levels of model genes.

### Correlation of Clustering Type and Immune Microenvironment

**FIGURE 6:**
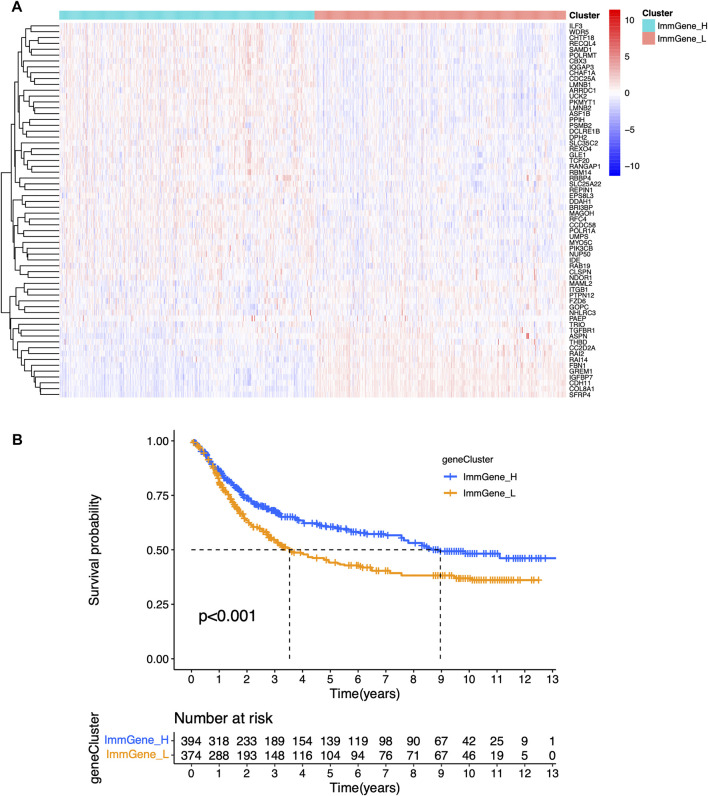
Sample clustering based on m6A regulatory genes **(A)** Clustering heat map **(B)** Survival curve.

**FIGURE 7:**
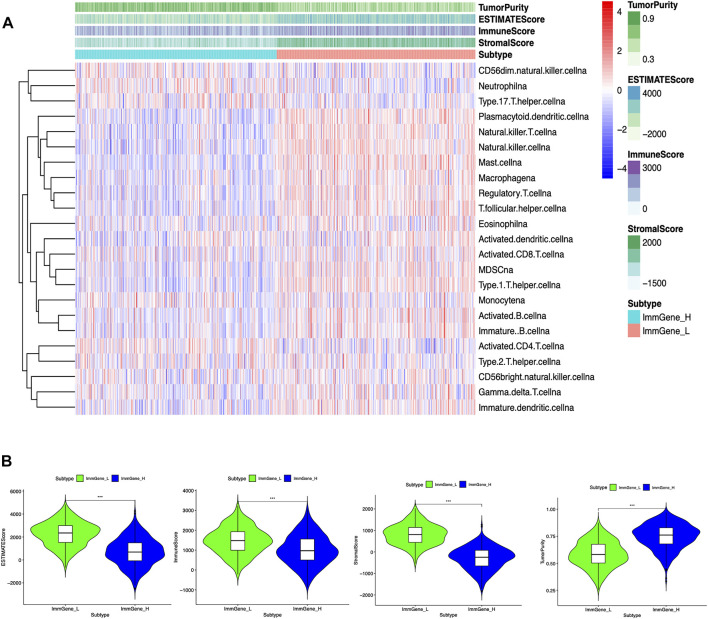
Associations of the tumor microenvironment with m6A regulatory genes **(A)** Heat map. The ordinate signifies the immune gene set and the abscissa represents the sample name **(B)** Violin plot. ***p < 0.001; **p < 0.01; *p < 0.05.

**FIGURE 8:**
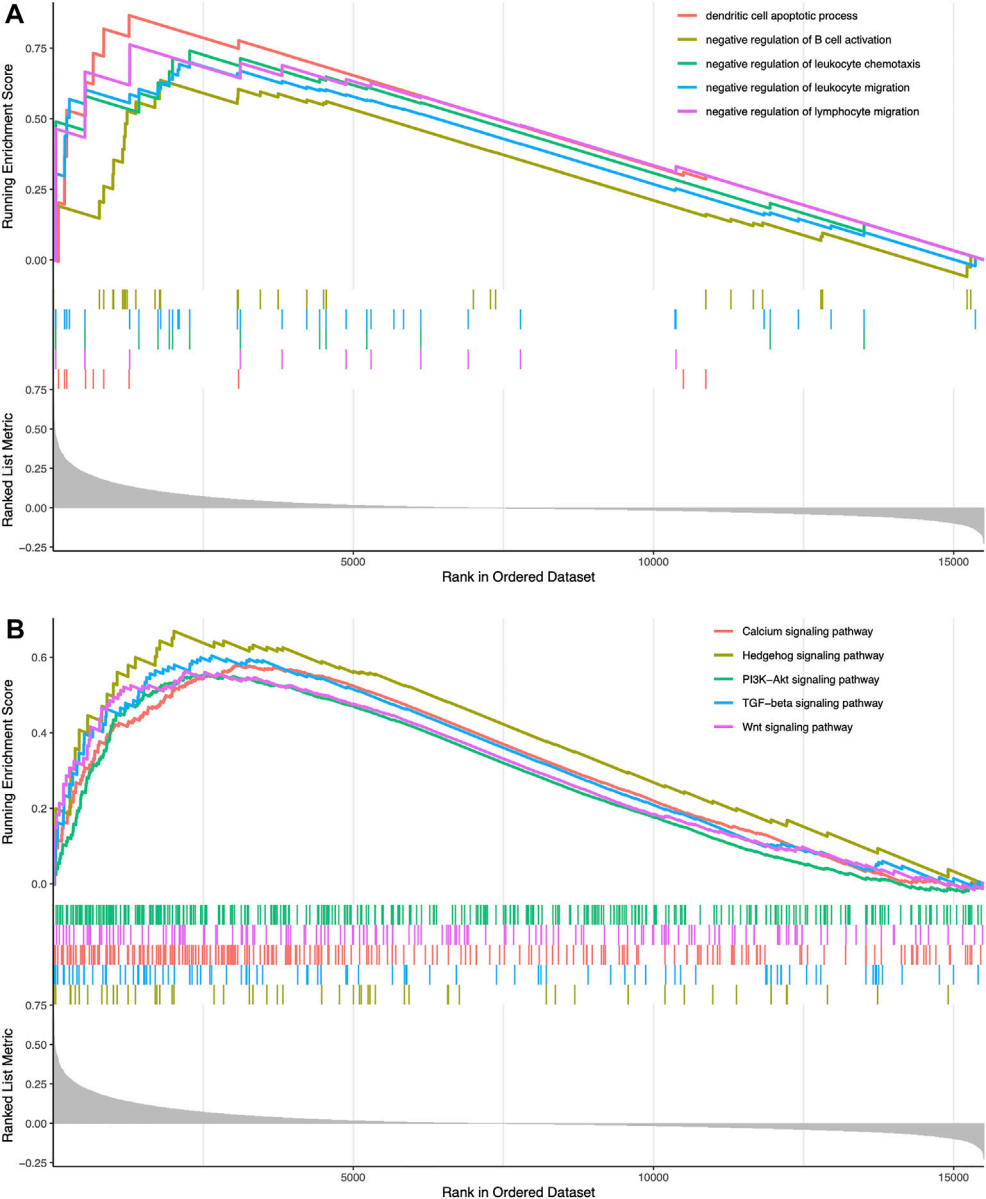
Enrichment-related analysis **(A)** In the immune gene high-expression group, GO enrichment results showing GO terms were obviously enriched **(B)** In the immune gene high-expression group, KEGG enrichment results showing its pathways were obviously enriched.

We classified TCGA samples into low-, and high-expression groups, and the classification is based on the prognostic impact of m6A-related immune genes. After our survival analysis study, these patients with high expression of m6A regulatory genes tended to have higher survival rates (Figure 6). The heat maps and violin plots of the tumor microenvironment showed that the m6A regulatory gene high-expression group had a higher tumor purity and lower level of immune cell infiltration in human tumors (Figure 7). The GSEA enrichment results reflected an important phenomenon in which low expression of immune genes was accompanied by the activation of an immunosuppressive function. This may be an important reason for that the low immune gene expression group showed a lower survival rate (Figure 8).

### Multi-Omics Analysis of Model Genes

Based on our prognostic model, we further compared the correlations of all model genes with stem cell indices and tumor microenvironment parameters. Initial findings indicated that RANGAP1, GOPC, NHLRC3, FZD6, and IDE were significantly correlated with stem cell indices and tumor microenvironment parameters (Supplementary Figure 1). It is visualized from the multi-omics results that the CNV frequency and mutation frequency of the model genes were very low (Supplementary Figure 2). In particular, CNV in all model genes was able to affect the standard in tumor-infiltrating immune cells in STAD. Interestingly, only the infiltration level of Macrophage could affect the survival rate of STAD (Supplementary Figure 3). In addition, structural domain mutations were present in FZD6, IDE, PAEP, RAB19, and RANGAP1 (Supplementary Figure 4).

### Drug Sensitivity Analysis

In the prognostic model, we performed drug sensitivity analysis separately for model genes and screened the top 16 drugs, and in the model genes, these drugs were highly correlated with gene expression (Supplementary Figure 5A). We used these stomach adenocarcinoma drugs, such as cisplatin, doxorubicin, gemcitabine, and lapatinib, to elucidate the prognostic model associated with m6A, and these analyses helped us understand the clinical value of STAD treatment. The results showed that gemcitabine was more sensitive in the high-risk group (Supplementary Figure 5B).

### Knockdown of RAB19’s Effects in Stomach Adenocarcinoma Cells Proliferation *in Vitro*

**FIGURE 9:**
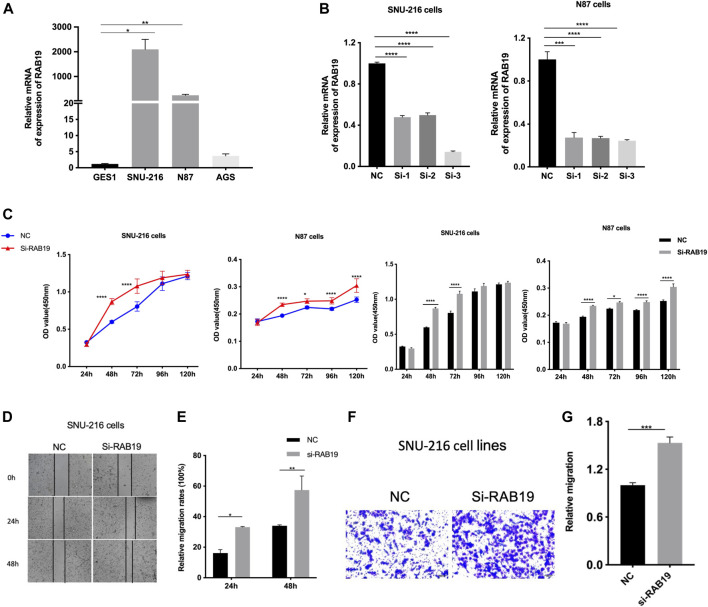
Knockdown of RAB19 promotes migration and proliferation of gastric tumor cells **(A)** In the STAD cell lines SNU-216, N87, AGS, and normal gastric epithelial cell line GES1, the relative expression level of RAB19 was detected by RT-PCR **(B)** In the SNU-216 and N87 cell lines, the transfection efficiency of si-RAB19 was detected by RT-PCR **(C)** In the SNU-216 and N87 cell lines, the effect of RAB19 knockdown on the proliferation was detected by the CCK-8 assay **(D)** Representative images from the wound healing assay **(E)** After knockdown of RAB19, the results of wound-healing assay were analysed **(F)** Representative images from the transwell assay **(G)** In the SNU-216 cell lines, the transwell assay results after knockdown of RAB19 were statistical analysed. ***p < 0.001, **p < 0.01, and *p < 0.05.

Firstly, we applied qRT-PCR to detect RAB19 mRNA expression, and in this study, SNU-216, N87 cells, and AGS cell lines were used. In our experiments, we obtained a higher expression of RAB19 in STAD cell lines SNU-216 and N87 than in normal gastric epithelial cells with GES1 (Figure 9A), and this is the reason why we selected them as subsequent knockdown experimental cell lines *in vitro*. In SNU-216 and N87 cells, RAB19 was knocked down. The material is siRNAs and transfection reagent. To ensure the reliability of the results, knockdown efficiency was examined by qRT-PCR (Figure 9B). We also performed CCK8 assays, an assay that explores the effects of RAB19 knockdown on the proliferation of STAD cells, and as a result, cells that underwent RAB19 knockdown (SNU-216 and N87 cells) had a more prominent proliferative capacity (Figure 9C). This was followed by the addition of wound-healing and transwell assays, which were used to investigate the effects of knockdown on cell migration. All experiments confirmed that knockdown increased the migratory capacity of SNU-216 cells (Figures 9D–G).

### Overexpression of RAB19’s Effects in Stomach Adenocarcinoma Cells Proliferation *in Vitro*

**FIGURE 10:**
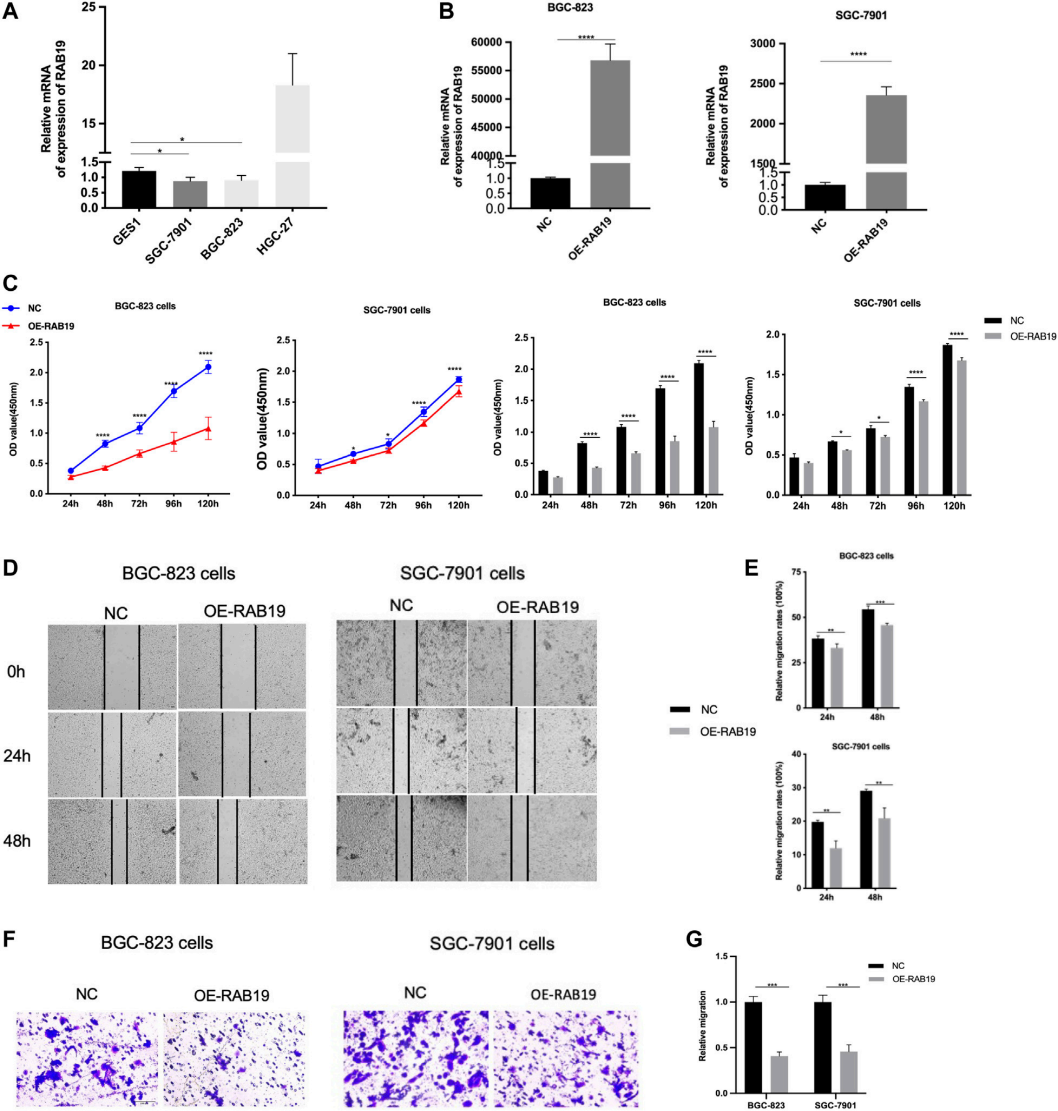
Overexpression of RAB19 inhibits migration and proliferation, in gastric tumor cells **(A)** In the STAD cell lines, SGC-7901, BGC-823, HGC-27, and normal gastric epithelial cell line GES1, the relative expression level was detected by RT-PCR **(B)** In the BGC-823 and SGC-7901 cell lines, the transfection efficiency of OE-RAB19 was detected by RT-PCR **(C)** In the BGC-823 and SGC-7901 cell lines, the effect of RAB19 overexpression on the proliferation was detected by the CCK-8 assay **(D)** Representative images from the wound healing assay **(E)** After overexpression of RAB19, the results of wound-healing assay were analysed **(F, G)** After overexpression of the RAB19, the transwell assay results were statistical analysed in the BGC-823 and SGC-7901 cell lines. ***p < 0.001, **p < 0.01, and *p < 0.05.

Then, with the same method (qRT-PCR assay), we proceeded to examine the expression of RAB19 mRNA in STAD cell lines, including HGC-27, BGC-823, and SGC-7901. We found that the RAB19 in STAD cell lines (SGC-7901 and BGC-823) was presenting lower expression than in GES1 (Figure 10A), and this is the reason why we selected them as subsequent overexpression experimental cell lines *in vitro*. The efficiency of their overexpression of RAB19 was verified through qRT-PCR (Figure 10B). CCK8 experiments showed that the proliferation (SGC-7901 and BGC-823 cells) was significantly decreased after RAB19 overexpression (Figure 10C). Subsequently, we also found that the migration ability was inhibited after RAB19 overexpression in these two cell lines (Figures 10D–G). Thus, RAB19 may be a protective factor that inhibits the migration and proliferation of STAD cells.

## Discussion

This discovery and comprehensive analysis of prognostic biomarkers can help clinicians to refine treatments and determine prognosis, as well as provides in-depth insights into the relevant pathophysiological mechanisms. Using various bioinformatics algorithms, we successfully developed a high-performance prognostic model based on the statistical correlations between m6A-regulated genes and immune genes, with a corresponding web-based dynamic nomogram APP that enhanced the practical and translational significance of the model. Further, our results revealed the correlations between two typologies with the tumor microenvironment and m6Ascore. Next, multi-omics analysis and drug sensitivity analysis targeting model genes enriched the study. Finally, knocking down and overexpressing RAB19 in STAD cell lines altered the proliferation and migration ability of the cells.

Research on big data provides unprecedented opportunities for biomedical development and enables to use many new technologies and methods for diseases treatment. For instance, gene therapy, gene diagnosis and targeted drugs. An increasing amount of clinical evidence demonstrates that in myeloproliferative neoplasms, advanced renal-cell carcinoma, gastric cancer, hepatocellular carcinoma, and aggressive prostate cancer, the combination of dependence on clinical data and genetic databases can provide individualized prediction of patient prognosis. It can also help in diagnosing and treating these patients (Grinfeld et al., 2018; Voss et al., 2018; Long et al., 2019; Kreuz et al., 2020; Zuo et al., 2020; Zhang et al., 2021a). Moreover, research in genetics has allowed us to better perform risk stratification of patients in clinical care (Nabors et al., 2020). In the case of STAD, a variety of prognostic models have been developed with wide application prospects (Shimura et al., 2022; Spolverato et al., 2022; Zhang et al., 2022). We have developed a high-performance prognostic model based on m6A-associated immune genes that has been successfully internally and externally validated and shows some degree of clinical translational value.

The model genes involved in this study, as high-risk independent prognostic factors for STAD, are currently available in published databases. The current study showed that RanGAP1 is dysregulated in a variety of cancers (Boudhraa et al., 2020). RANGAP1 is a nuclear transport protein, that is one of the components of the RanBP2 subcomplex, and the SUMO-ization of RanGAP1 is essential for its nuclear pore localization (He et al., 2021). In addition, RNA interference with RanGAP1 enhances DLBCL cell death and cell cycle arrest (Chang et al., 2013). It was found that the GTPase activity of Ras-related Ran can be induced by RanGAP (Bischoff et al., 1994). Ran expression has been verified to be higher in kidney, breast, colon, gastric, pancreatic, lung, and ovarian cancers than in normal groups (Abe et al., 2008; Barrès et al., 2010; Sheng et al., 2018; Lu et al., 2020). Moreover, Ran has been verified to be linked to higher grade, metastasis and local invasion in kidney, ovarian and breast cancers, and gastric cancer (Boudhraa et al., 2020; Lu et al., 2020). NHLRC3, a member of the miR-93-5p/17-5p/NHLRC3 axis, is also implicated in the progression of colon cancer, and it has even been reported to be an independent prognostic marker for colorectal cancer and a possible target for cancer therapy (Chen et al., 2017; Yang et al., 2022). Additionally, some recent study showed using bioinformatics analysis that NHLRC3 correlates extremely well with the prognosis of gastric adenocarcinoma, and it play an important role the development of stomach cancer (Xu et al., 2016). FZD6, a member of the frizzled family, is a receptor for Wnts. There are numerous studies show that FZD6 expression is associated with the malignancy and prognosis of breast cancer, cervical cancer and human glioblastoma (Zhang et al., 2021b; Wang et al., 2021; Assidi et al., 2022). FZD6 also can inhibit the migration and proliferation of gastric cancer cells *via* targeting miR-21 to activate the non-classical Wnt pathway (Yan et al., 2016; Ko et al., 2022). The current research on RAB19 is not very clear. Some studies have reported that in colorectal cancer, RABs may play an important role in regulating cell cycle and immune-related pathways, and in gastric cancer, the miR495-5p/RAB19 axis inhibits its proliferation, migration, and invasion, so RAB19 perhaps be a potential biomarker for predicting the prognosis and immunotherapeutic response of related tumors (Hu and Ji, 2021; Jewett et al., 2021; Jiang et al., 2022). GOPC, a protein that containing a coiled helix motif, is also one of the prognostic markers of Early-Stage Lung Squamous Cell Carcinoma, and when fused with ROS1 and other ROS1, it can represent a rare but recurrent drug target in various glioma types (Li et al., 2021; Sievers et al., 2021). PAEP, a glycodelin gene, is connected with immune infiltration. It was identified in one study as an independent significant risk factor for Clear Cell Renal Cell Carcinoma (ccRCC) and as one of the survival predictive prognostic markers for melanoma (Sawyer, 2021; Wu et al., 2021; Zeng et al., 2022). IDE is a multifunctional protease whose main therapeutic areas are neuronal diseases and metabolism. However, the recent reports have indicated that it may be a potential target for cancer (Lesire et al., 2022). Actually, studies on the role of GOPC, PAEP and IDE in STAD are mostly lacking, but they all have important roles in other cancer types. Therefore, more preliminary single-gene bioinformatics analysis is needed to validate their functions in STAD (Yfanti et al., 2008; Dydensborg et al., 2009; Ohara et al., 2017). These previous studies demonstrate, from a different perspective, that the inclusion of these seven modular genes in our signature is reasonable and reliable. In addition, our results suggest that RAB19, GOPC, PAEP, and IDE may be novel prognostic predictive genes for STAD. Furthermore, our study has shown that interfering with RAB19 expression improved the capacity of cell migration and cell proliferation and (SNU-216 and N87) *in vitro*, which was inversely validated by overexpression of RAB19, indicating the importance and potential role of RAB19 in gastric adenocarcinoma. However, more researches are needed to further explore the specific mechanisms especially with RAB19 inhibits tumorigenesis development in STAD cells. Furthermore, the biofunction of seven prognosis-related m6A-associated immune genes in gastric adenocarcinoma remains largely unknown, with further studies on the effects of model genes on gastric adenocarcinoma needed. Future studies may focus on exploration of their potential mechanisms in STAD through molecular functional assays.

Based on the ssGSEA results and clustering grouping, the immune gene low-expression group accompanied by lower survival rates and a higher immune cell infiltration. In the tumor microenvironment, the multi-GSEA enrichment map provides a possible explanation for this higher degree of immune infiltration, which is accompanied by enhanced immunosuppressive activity, involving extensive regulation of multiple immune cell biological behaviors and multiple signaling pathways. Thus, it ultimately leads to a lower survival rate of patients.

Recent studies on m6A have revealed that it has key roles in various cancers. Inhibition of m6A-associated proteins effectively attenuates tumor cell self-renewal and overcomes methylating-agent-induced immune evasion, which provides an opportunity to develop effective targeted therapies (Su et al., 2020). Bo Zhang et al. showed that the effective immune infiltration was lacking in high m6Ascore subtypes in STAD, and it was correlated with poor survival (Zhang et al., 2020). *In vitro*, m6A protein knockdown sensitized melanoma cells to IFNγ to promote to the result of mouse’s anti-PD-1 inhibitors (Yang et al., 2019; Huang et al., 2020). Although there have been fewer m6A-related studies in STAD, the low m6A-related phenotype may provide obvious clinical benefits and therapeutic advantages. And in order to promote the efficacy of immunotherapy, we enhance the response to anti-PD-1/L1 immunotherapy and increase STAD neoantigen load (Zhang et al., 2020).

In combination with the co-expression network described above, these findings indicate that m6A-regulated genes and immune genes have extensive interactions in STAD and are closely associated with the suppression of immune activity. Thus, they are of biological importance.

In addition, in the high- and low-risk groups, the mutational load results were used to refine the survival stratification of STAD participants; this will help to determine the prognosis of patients more precisely. Also, multi-omics analysis of model of genes suggested that the expression levels of some model of genes and CNV could themselves influence immune cell infiltration in STAD. From the above, we can see the comprehensiveness and complexity of the factors regulating the tumor microenvironment.

## Conclusion

In summary, we have constructed a high-performance prognostic model for STAD and a corresponding web-based dynamic nomogram APP that enhances the model’s clinical significance. In addition, the correlations of Clustering Analysis and the tumor microenvironment provided further insight into the pathophysiological process of STAD. These results will help to improve the accuracy of prognosis of STAD patients, as well as providing new ideas and inspiration for more in-depth mechanistic studies. What’s more, *in vitro*, interference of RAB19 was shown to promote invasion and migration of gastric adenocarcinoma cells, while its overexpression inhibited these functions, suggesting a potential therapeutic role for RAB19 in stomach adenocarcinoma.

## Data Availability

The datasets presented in this study can be found in online repositories. The names of the repository/repositories and accession number(s) can be found in the article/Supplementary Material.

## Abbreviations

AUC, Area under the curve; GEO, Gene Expression Omnibus; GO, Gene Ontology; GSEA, Gene Set Enrichment Analysis; KEGG, Kyoto Encyclopedia of Genes and Genomes; LASSO, Least absolute shrinkage and selection operator; ROC, Receiver operating characteristic; ssGSEA, Single Sample Gene Set Enrichment Analysis; TCGA, The Cancer Genome Atlas.
