# Supplementary material for: Prognostic Role of M6A-Associated Immune Genes and Cluster-Related Tumor Microenvironment Analysis: A Multi-Omics Practice in Stomach Adenocarcinoma
Source: Front Cell Dev Biol. 2022 Jun 24;10:935135. doi: 10.3389/fcell.2022.935135 (PMC9291731; doi:10.3389/fcell.2022.935135)
Supplement: Supplementary file 1 [file Table1.DOCX]

<https://www.jianguoyun.com/p/DWVF_KIQruCvCBi_jqwE>

STAD row data

<https://www.jianguoyun.com/p/DVsSL-YQruCvCBinyKwE>

RAB19 experimental data
