# Supplementary material for: Prognostic Role of M6A-Associated Immune Genes and Cluster-Related Tumor Microenvironment Analysis: A Multi-Omics Practice in Stomach Adenocarcinoma
Source: Front Cell Dev Biol. 2022 Jun 24;10:935135. doi: 10.3389/fcell.2022.935135 (PMC9291731; doi:10.3389/fcell.2022.935135)

Supplementary Figure 1

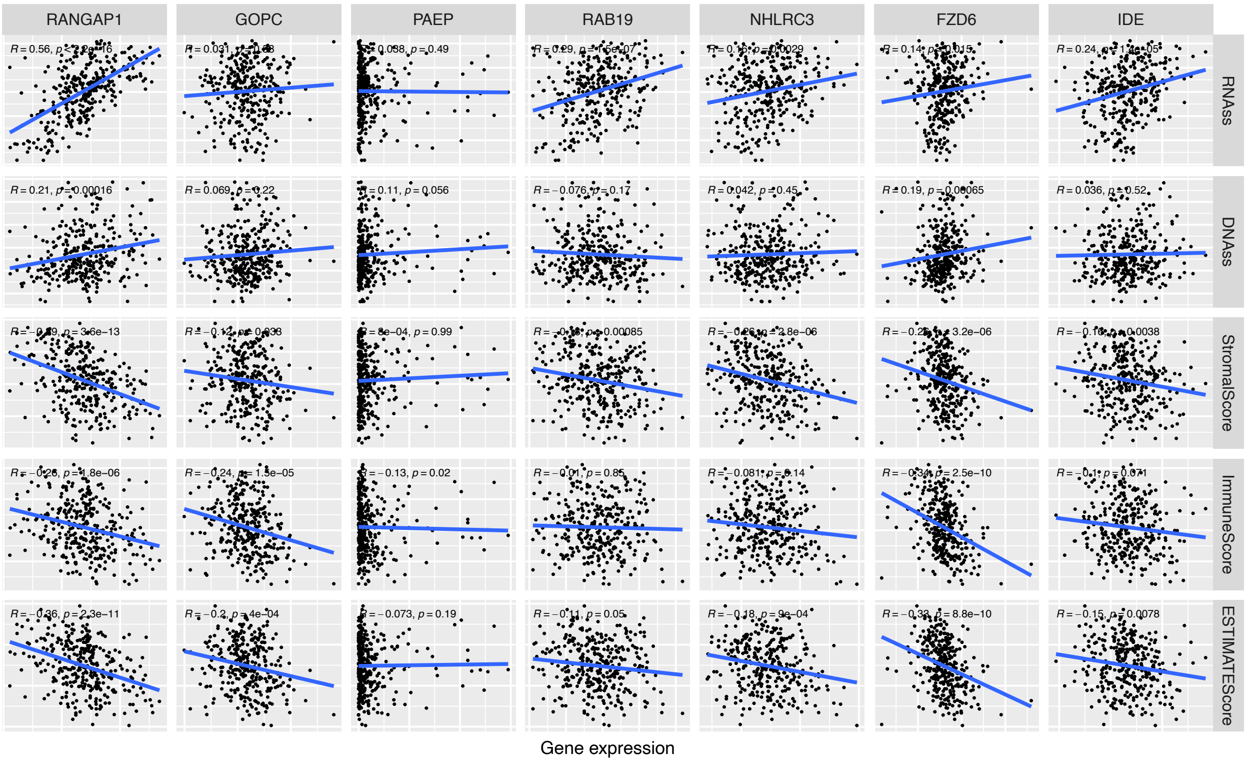

Supplementary Figure 2

A

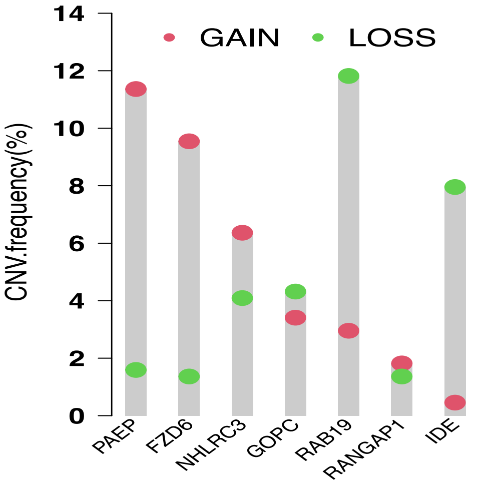

B

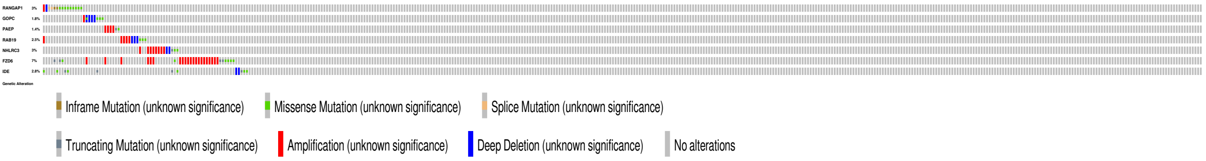

Supplementary Figure 3

A

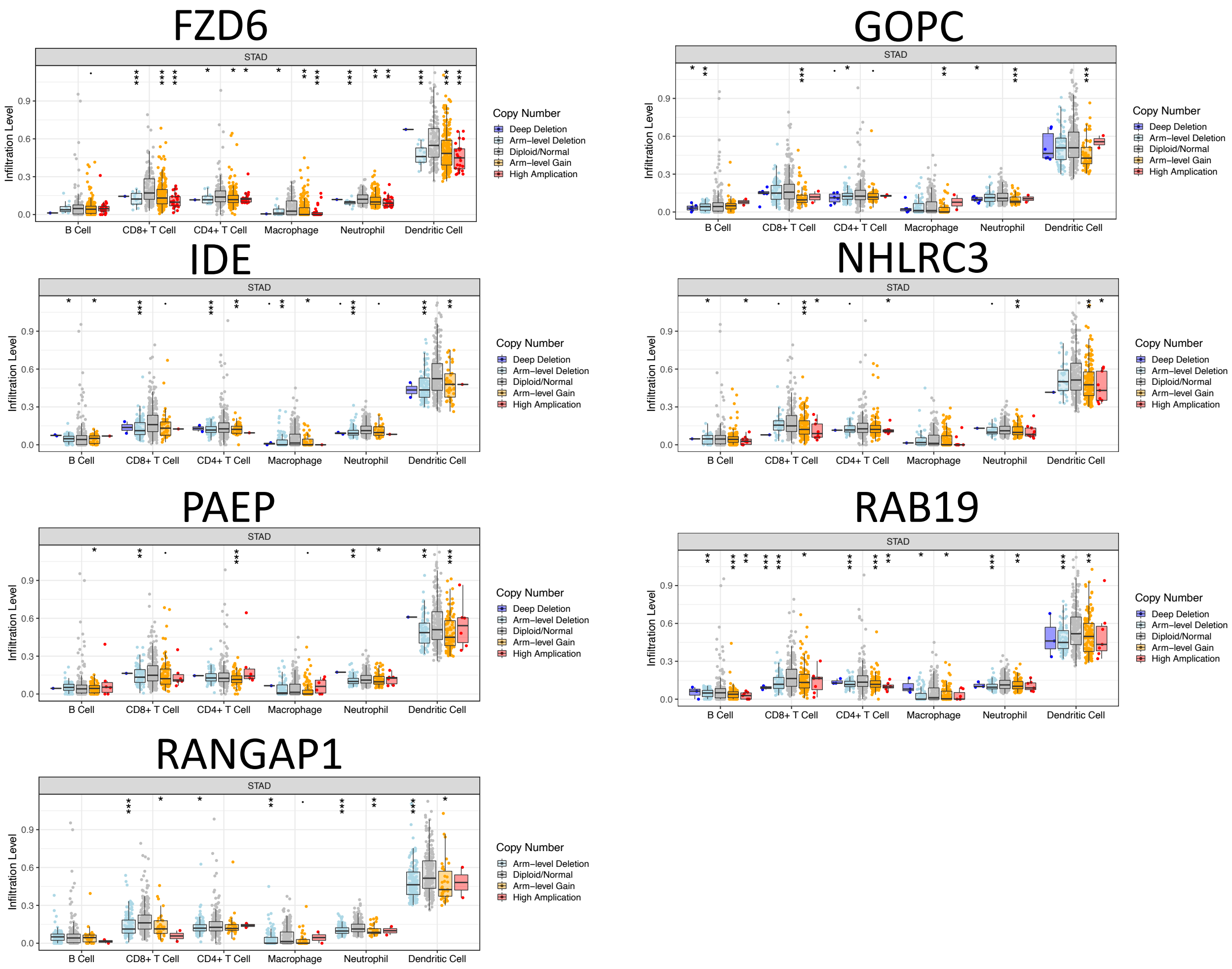

B

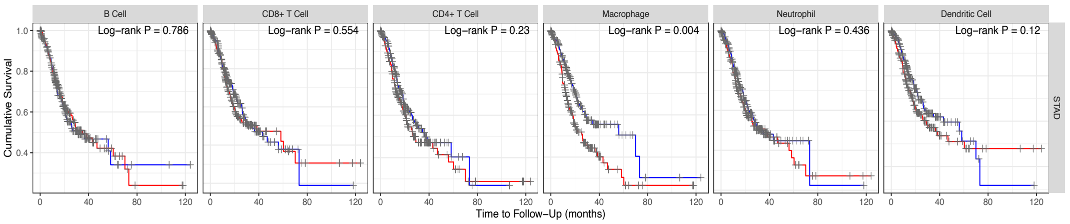

Supplementary Figure 4

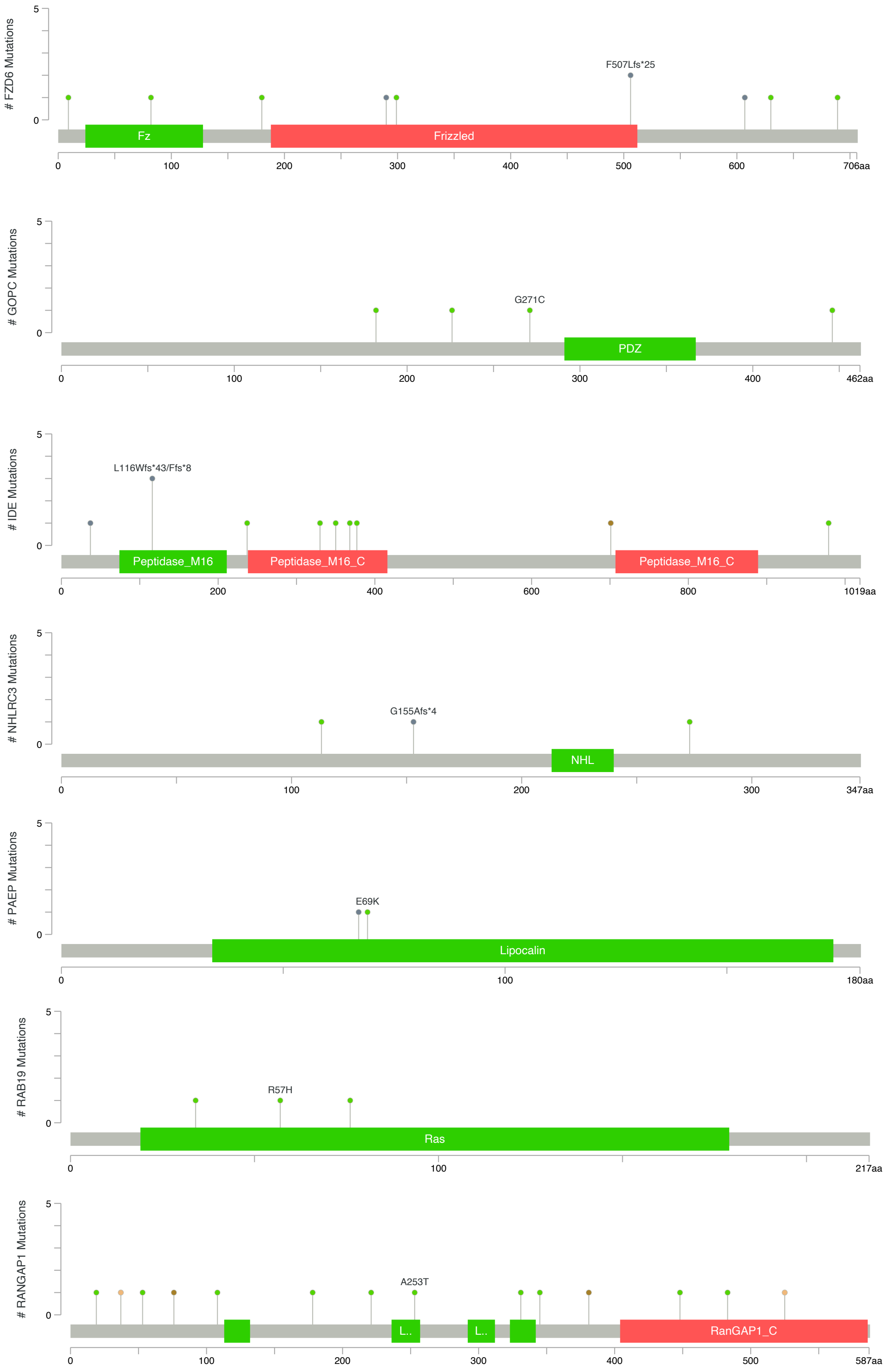

Supplementary Figure 5

A

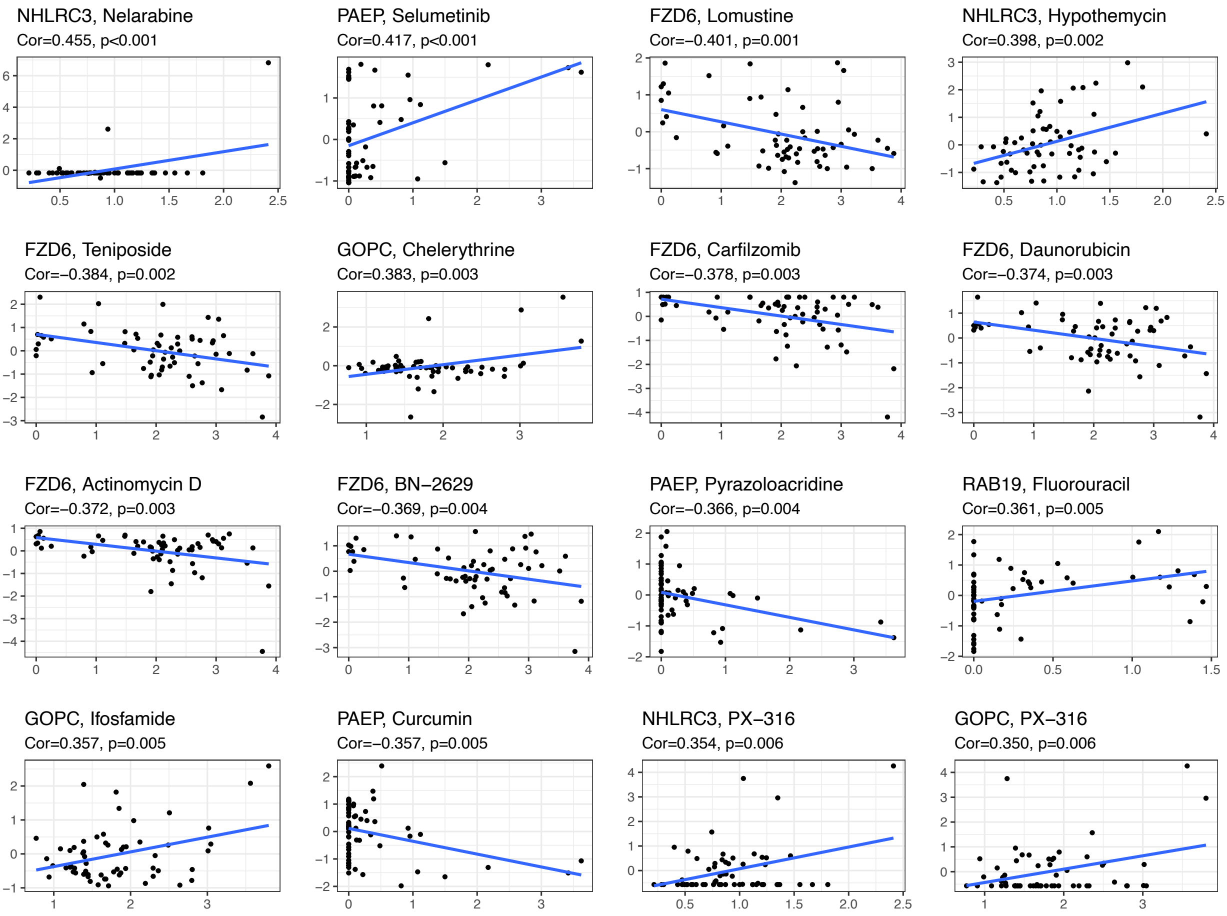

B

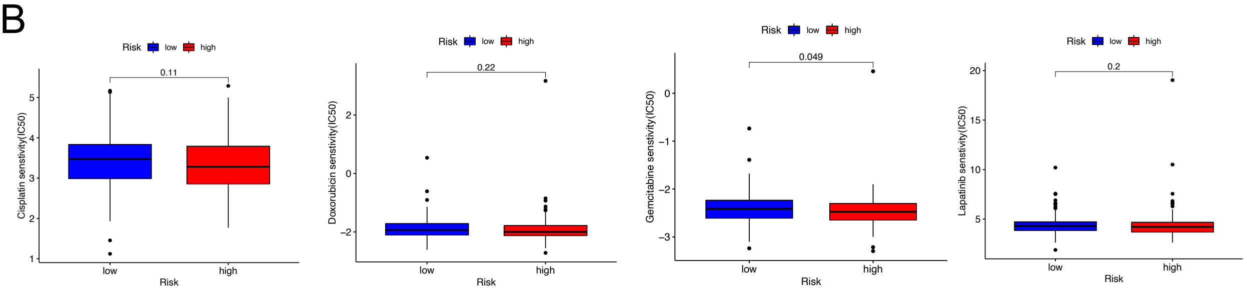

Supplement: Supplementary file 2 [file DataSheet1.PDF]
